# Bisphosphonates and Risk of Upper Gastrointestinal Cancer — A Case Control Study Using the General Practice Research Database (GPRD)

**DOI:** 10.1371/journal.pone.0047616

**Published:** 2012-10-24

**Authors:** Ellen Wright, Peter T. Schofield, Paul Seed, Mariam Molokhia

**Affiliations:** Department of Primary Care and Public Health Sciences, King's College London, London, United Kingdom; University of Aberdeen, United Kingdom

## Abstract

**Background:**

Concerns have been raised as to the safety of bisphosphonates; in particular a possible link between bisphosphonate use and upper gastrointestinal (GI) cancer. Two published studies using different study populations but drawn from earlier versions of the same national UK database, reached differing conclusions: one finding no evidence for an increase in the risk of gastric or oesophageal cancer in bisphosphonate users and one finding a small but significantly increased risk of oesophageal cancer linked to duration of bisphosphonate use.

**Methodology/ Principal Findings:**

Design-A case control study comparing bisphosphonate prescribing in cases of upper GI cancer from 1995 to 2007 using UK primary care electronic health records (GPRD).

Main Outcome Measure-Relative Risk (approximated to Odds Ratio for rare events) for oesophageal and gastric cancer development in bisphosphonate users compared to non–users. The odds of being a case of oesophageal cancer, adjusted for smoking status, were significantly increased in women who had had one or more bisphosphonate prescriptions, odds ratio 1·54 (95% CI 1·27–1·88) compared to non-users. There was no significant effect on gastric cancer in women, odds ratio adjusted for smoking status, 1.06 (95% CI 0.83–1.37) and also no apparent risk in men for either oesophageal or gastric cancer, odds ratio adjusted for smoking status 0.78 (95%CI 0.56–1.09) and 0.87 (95% CI 0.55–1.36) respectively.

**Conclusions/ Significance:**

Our results support a small but significant increased risk of oesophageal cancer in women prescribed bisphosphonates and is based on the largest number of exposed cases to date in the UK.

## Introduction

There has been a 20-fold rise in the prescribing of bisphosphonates in general and alendronate in particular in recent years. In 1992 0·2% of women over 40 included in the UK General Practice Research Database (GPRD) were prescribed a bisphosphonate but by 2005 this had risen to 4·1%. At the same time there has been a parallel reduction in hormone replacement therapy (HRT) prescribing from 8·2% in 1991 to 7·0% in 2005, with a 50% fall since 2002, largely driven by concerns over excess risk of breast cancer (and to a lesser degree ovarian cancer) and cardiovascular events.[1]

Bisphosphonates and particularly alendronate are well known to cause both dyspepsia and inflammatory changes such as erosive oesophagitis, delayed healing, and mucosal abnormalities.[2] Multinucleated giant cells have been detected in oesophageal inflammatory exudates. Whether these can undergo malignant transformation is not known but concerns have been raised as to a possible link between bisphosphonate use and upper GI cancer.[3], [4]

Wysowski[3], in her report to the US Federal Drug Administration (FDA), noted that since the initial marketing of alendronate in 1995 the FDA had received 23 reports of patients who developed oesophageal tumours after taking the drug. Typically 2 years elapsed between the time patients started taking the drug and the onset of oesophageal cancer. In Europe and Japan a further 31 cases had been reported linking oesophageal cancer and bisphosphonate use. The FDA reporting did not include any denominator data. Wysowski suggested distal oesophageal carcinoma might be associated with bisphosphonate use but recommended more rigorous study approaches with sufficient size, length of follow up, inclusion of a control group, and control for confounding variables. In the meantime the authors advised that the drugs should not be used in patients with Barrett's oesophagus (an abnormal change in the cells of the lower portion of the oesophagus, thought to be due mainly to chronic acid reflux from the stomach, its main significance is an increased risk of developing adenocarcinoma of the oesophagus). However, Merck (the makers of alendronate) have not reported any cases of oesophageal cancer linked to bisphosphonate use in their clinical database of 17,000 patients.

Following the FDA reports, rapid communications of studies using a large national database in Denmark and Medicare beneficiaries in the US concluded there was no evidence for an increased risk of oesophageal cancer in bisphosphonate users[5], [6]. However, again the follow up period was short (2 years). Because of the relative rarity of these conditions and the limited number of patients, the confidence intervals were wide and it was impossible to conclude whether there could be a clinically important association. Two published studies using the UK General Practice Research Database (GPRD) reached differing conclusions. Cardwell et al.[7] in a retrospective cohort study found no evidence of an increase in the combined risk for gastric and oesophageal cancer in bisphosphonate users compared to non-users, Hazard Ratio 0·96, (95% CI 0·74–1·49) but again confidence intervals were wide (i.e. study is also consistent with a fairly substantial effect). Green et al.[8] carried out a nested case control study using a sample drawn from the same database and found an overall increased risk of oesophageal cancer in bisphosphonate users (Relative Risk 1·30, 95% CI 1·02–1·66). This increased with more than ten prescriptions or longer than three years use (RR 1·93). Although these studies appear to give different results, the relatively wide confidence intervals overlap substantially, so results could be consistent with a similar magnitude of risk. In Cardwell's study a relatively small proportion of exposed cases were included limiting power and precision.

Dixon and Solomon [9] have reviewed in detail the conflicting results of these two studies and concluded similarly that even when confining the comparison to patients with greater than three years exposure to bisphosphonates (allowing for induction and latency periods) the confidence intervals of the relative risks for both studies included the possibility of a 50% increase in risk. At less than three years exposure, although neither study found an increase in relative risk of oesophageal cancer with bisphosphonate use, Dixon and Solomon point out that the confidence intervals of both span one and, therefore, one should say that the results are ‘inconclusive’ rather than “there is no effect”. The upper limits of the RR's of 3 years' exposure are 1.73 and 1.81 respectively according to the 95% confidence intervals of the two analyses. They note “it is, therefore, plausible in both studies that, despite the best guess being of ‘no increased risk’, there may be as much as a 70% increase in baseline risk.”

Haber et al[10] concluded after reviewing both Green and Cardwell's studies and the observational study on the incidence of oesophageal cancer in patients with Barrett's oesophagus taking bisphosphonates by Nugyen[11], as well as the case reports in Wysowski's article, that “the evidence on the use of bisphosphonates and risk of esophageal cancer is weak and conflicting”.

Our primary aim was to carry out a retrospective case control study with the greatest possible power using a large UK primary care database (the GPRD), to determine whether any association exists between prescribing of alendronate specifically (and bisphosphonates in general) and the development of upper GI malignancy. We were particularly concerned to have enough statistical power to detect a small increase in a rare, but serious disease and, therefore, chose to do a matched case control study using all known cases of oesophageal and gastric cancer. Although this is the third study using data drawn from the GRPD, it uses a later and significantly larger version of the database and it is substantially more powerful.

## Methods

### Source population

The study population included all adults (men & women) registered with up to standard GP (General Practitioner) practices in the UK General Practice Research Database (GPRD) from 1/1/1995 to 31/12/2007. The GPRD is a primary care database holding approximately 5 million longitudinal anonymised records of patients registered with a national health service (NHS) GP.[12] Only UK general practices with data of approved quality can contribute to the database. Strict protocols are followed by practices for data entry. The accuracy and completeness of GPRD data, particularly with respect to prescribing (almost 100%) and cancer diagnosis (95%) is high and has been confirmed in validation studies.[13], [14]. The data extraction was performed from the May 2010 version of the GPRD.

Case and control definition

Cases had a clinical or referral record of incident upper GI (UGI) malignancy in the study period and their registration period.

UGI cancer events were identified by the following READ codes: (See appendix S1 for full list)

B11 + all daughter codes–malignant neoplasm of stomach

B10 + all daughter codes–malignant neoplasm of oesophagus

BB55–linitis plastica

BB5C–gastrinoma and carcinoma

B105–malignant neoplasm of lower third of oesophagus

B10z–oesophageal cancer

B11-1–gastric neoplasm

For every case, four control patients matched on age (+/−2 years) and gender and with no record of UGI cancer were selected from the database. Observation window matching was used: i.e. observation of all four controls started before that of the case, and ended after that of the case. The observation times of the controls matched exactly those of their matched cases. Control events outside the observation window of the case were ignored. The period of follow up was earliest of: death, last medical record, practice transfer, or end of study period.

Exposure assessment. (bisphosphonate use)

Bisphosphonate use was defined as any patient ever prescribed a bisphosphonate during the study period. We excluded patients with prescriptions for bisphosphonates licensed to treat Paget's disease or bone metastases (pamidronate, ibandronate) in the sensitivity analysis as these patients would already be suffering from cancer or Paget's disease. Duration of bisphosphonate use was the time between first and last prescription. We categorized bisphosphonate use by number of prescriptions issued in the study period into low (<10 prescriptions) and high (>10 prescriptions).

### Covariates

As covariates and potential confounders we evaluated the presence of smoking as a major risk factor for UGI cancer. Smoking was defined as any record of use from 1980. We also adjusted for alcohol intake, dyspepsia, proton pump inhibitor (PPI) use, *Helicobacter pylori* (*H. pylori*) status and body mass index (BMI) although previous studies had not shown an effect.[8]

Sample size calculation

Typically for rare diseases case control studies are used to maximize efficiency and power to provide a valid estimate of risk or hazard ratio. Using multiple controls per case can increase power, although the gain in power beyond 4 controls is relatively small. With 8,000 cases & 32,000 controls, we calculated we would have 89% power to detect a difference of 2% (50% compared to 52%) in the rate of bisphosphonate use in the two arms. If the rate of bisphosphonate use was lower, the minimum detectable odds ratio would be increased; but its clinical importance would be similar. For example, for 4% use in controls (a more realistic figure based on recent figures) [8] and 4·8% use in cases, we would be able to detect an odds ratio of 1·21 with 88% power. We used a matched (age and gender) design to reduce confounding.

### Statistical methods

Taking into account the matched study design we used conditional logistic regression to calculate unadjusted and adjusted odds ratios (ORs) and corresponding 95% confidence intervals (CIs) for the association between bisphosphonate use and UGI cancer. We adjusted for the main confounder smoking, as well as alcohol intake, dyspepsia, proton pump inhibitor (PPI) use, *H. pylori* status and body mass index (BMI). We tested for the interaction between gender and bisphosphonate use and risk of upper GI cancer. We restricted analyses to oesophageal cancer, gastric cancer and alendronate alone. We carried out sensitivity analyses as follows: excluding bisphosphonates used to treat bone metastases; excluding bisphosphonates commenced 6 months or less before the diagnosis of UGI cancer and excluding cases where the READ code for UGI cancer was uncertain. All analyses were conducted using Stata version 11 (Stata Corporation, USA).

## Results

We received data on 8,636 cases of UGI cancer where the cancer diagnosis date was between January 1, 1995, and December 31, 2007, and 34,544 controls, 4 per case, matched on age (+/−2 years) and sex. We initially analyzed the complete data set and then looked at the subsets of oesophageal and gastric cancer cases and controls. Table 1 gives summary descriptive statistics separately for the cases and controls.

**Table 1:** Summary descriptive statistics for cases and controls.

Characteristics	OES CA CASES'	OES CA CONTROLS	GASTRIC CANCER CASES	GASTRIC CANCER CONTROLS	All UGI CA CASES	All UGI CA CONTROLS
Male	3,412	13,648	2,084	8,336	5,496	21,984
Female	1,814	7,256	1,326	5,304	3,140	12,560
Total (male and female)	5,226	20,904	3,410	13,640	8,636	34,544
Mean Age (at time of diagnosis of case)	63·4	63·4	66.5	66.5	64·7	64·7
Mean age men	61·6	61·6	65.6	65.6	63·1	63·1
Mean age women	67·0	67·0	68.1	68.1	67·5	67·5
Mean years observed in data base	6·3	6·3	5.7	5.7	6·1	6·1
Smoking-any positive record of smoking (%)	2,229 (51.5)	8,437 (45.4)	1,303 (46.5)	5,274 (43.9)	3,532 (49.5)	13,711 (44.9)
Dyspepsia–any record up to 12 mths before cancer diagnosis in cases and equivalent date in controls (%)	1,169 (22.3)	4,184 (20.0)	827 (24.3)	2,910 (21.3)	1,996 (23.1)	7,094 (20.5)
PPI-any use within study window (%)	1,100 (21.1)	3,215 (15.4)	728 (21.4)	2,073 (15.2)	1,828 (21.2)	5,288 (15.3)
*H. pylori* positive–any record	121 (2.3)	491 (2.4)	128 (3.8)	321 (2.4)	249 (2.9)	812 (2.4)
BMI>30 (%)	88 (1.7)	1,621 (7.8)	58 (1.8)	948 (6.7)	146 (1.7)	2,569 (7.44)
Alcohol intake–any record greater than recommended limits (%)	620 (11.9)	2,293 (11.0)	286 (8.4)	1,345 (9.9)	906 (10.5)	3,638 (10.5)
Prescribed bisphosphonate-male and female (%)	225 (4·3)	749 (3·6)	117 (3.4)	478 (3.5)	342 (4·0)	1227 (3·6)
Men (%)	54 (1.6)	246 (1.8)	24 (1.2)	124 (1.5)	78 (1.4)	370 (1.7)
Women (%)	171 (7.8)	503 (6.9)	93 (7.0)	354 (6.7)	264 (8.4)	857 (6.8)
Prescribed alendronate (%)	119 (2·3)	410 (2·0)	72 (2.1)	252 (1.9)	191 (2·2)	662 (1·9)
Men (%)	24 (0.7)	122 (0.9)	14 (0.7)	68 (0.8)	38 (0.7)	190 (0.9)
Women (%)	95 (5.2)	288 (4.0)	58 (4.4)	184 (3.5)	153 (4.9)	472 (3.8)
Prescribed bisphosphonate–xcluding those used to treat metastases (%)	218 (4·2)	726 (3·5)	113 (3.3)	461 (3.4)	331 (3·8)	1,187 (3·5)
Prescribed bisphosphonates-less than 10 prescriptions-mean number per time observed in database	3·8	4·0	3.9	3.8	3·8	3·9
Prescribed bisphosphonates–more than 10 prescriptions–mean number per time observed in database	26·5	26·2	23.0	26.0	25·4	26·1

## Statistical Analysis

### Risk of all bisphosphonates on UGI cancer

We initially investigated the effect of all bisphosphonates on UGI cancer in both men and women and found an Odds Ratio (OR) of 1·13 (95% CI 0·99–1·28) for men and women combined (appendix S2). The OR for the effect of all bisphosphonates on UGI cancer in women adjusted for smoking status was 1·34 (95% CI 1·14–1·56) and 0·81 (95% CI 0·62–1·06) for men.

### Risk of all bisphosphonates on oesophageal cancer and gastric cancer

When we analysed the effects of all bisphosphonates on oesophageal cancer only we found an OR of 1·43 (95% CI 1·18–1·72) in women and 0·87 (95% CI 0·65–1·18) in men. The corresponding results for gastric cancer were OR 1.06 (95% CI 0.83–1.35) in women and OR 0.77 (95% CI 0.50–1.20) in men. Adjusting the analyses for smoking status did not change the results significantly. The OR for the effect of all bisphosphonates on oesophageal cancer adjusted for smoking status was 1·54 (95% CI 1·27–1·88) for women and 0·78 (95% CI 0·56–1·09) for men (tables 2 and 3).

**Table 2:** Risk of bisphosphonates on oesophageal and gastric cancer in women.

	Oesophageal Cancer Odds Ratio (95% CI)	Gastric Cancer Odds Ratio (95% CI)	All UGI Cancer Odds Ratio (95% CI)
Women taking bisphosphonates	1.43 (1.18–1.72) (171 cases, 503 controls)	1.06 (0.83–1.35) (93 cases, 354 controls)	1·27(1.10–1.47) (264 cases, 857 controls)
Adjusted for smoking	1.54 (1.27–1.88)	1.06 (0.83–1.37)	1.34 (1.14–1.56)
Adjusted for smoking, alcohol intake, PPI, *H. pylori* status, dyspepsia andBMI	1.43 (1.16–1.75)	0.98 (0.76–1.27)	1.24 (1.06–1.45)

**Table 3:** Risk of bisphosphonates on oesophageal and gastric cancer in men.

	Oesophageal Cancer Odds Ratio (95% CI)	Gastric Cancer Odds Ratio (95% I)	All UGI Cancer Odds Ratio (95%CI)
Men taking bisphosphonates	0.87 (0.65–1.18) (54 cases, 246 controls)	0.77 (0.50–.20) (24 cases, 124 controls)	0·84 (0.66–1.07) (78 cases, 370 controls)
Adjusted for smoking	0.78 (0.56–1.09)	0.87 (0.55–1.36)	0.81 (0.62–1.06)
Adjusted for smoking, alcohol intake, PPI, *H. pylori*, dyspepsia andBMI	0.73 (0.53–1.03)	0.77 (0.49–1.21)	0.75 (0.57–0.98)

### Risk of alendronate alone on oesophageal cancer

When we restricted to alendronate alone we found corresponding ORs of 1.37 (95% CI 1.07–1.75) for women and 0.78 (95% CI 0.50–1.22). The OR for alendronate alone on oesophageal cancer adjusted for smoking status was 1·42 (95% CI 1·10–1·83) for women and 0·73 (95% CI 0·46–1·17) for men (table 4).

**Table 4:** Effect of alendronate on oesophageal cancer for men and women (mean number of observations 19,991.

	Odds Ratio	P value	95% confidence interval
Women on alendronate (95 cases, 288 controls)	1·37	0·014	1·07–1·75
Adjusted for smoking	1·42	0·007	1·10–1·83
Men on alendronate (24 cases and 122 controls)	0·78	0·28	0·50–1·22
Adjusted for smoking	0·73	0·19	0·46–1·17

### Interaction of bisphosphonate use and oesophageal cancer risk with gender

We looked at the interaction of bisphosphonate use and oesophageal cancer risk with gender, which gave an OR of 1·27 (95% CI 1·10–1·47) for women (Table 2) and 0·84 (95% CI 0·66–1·07) for men (Table 3); in other words there appeared to be an effect in women but not in men. The interaction between bisphosphonate use and gender, using the likelihood-ratio test (LRT) was statistically significant, p = 0.0011.

### Sensitivity analyses

We performed three sensitivity analyses none of which significantly altered the results (table 5). Firstly we excluded bisphosphonates licensed to treat bone metastases, as these patients by definition would already be suffering from cancer. The corresponding ORs for the effect of all bisphosphonates on UGI cancer adjusted for smoking status were 1·34 (95% CI 1·14–1·57) for women and 0·80 (95% CI 0·61–1·05) for men. Secondly we excluded prescriptions of bisphosphonates commenced six months or less before the UGI diagnosis date on the grounds that the time interval was too short for bisphosphonates to be causative. The subsequent ORs adjusted for smoking status were 1·30 (95% CI 1·10–1·53) for women and 0·77 (95% CI 0·58–1·04) for men. Finally we excluded cases where the READ code for UGI cancer diagnosis was uncertain. The ORs adjusted for smoking in these cases were 1·34 (95% CI 1·14–1·56) for women and 0·81 (95% CI 0·62–1·06) for men.

**Table 5:** Sensitivity Analyses: excluding bisphosphonates used to treat bone metastases; excluding prescriptions for bisphosphonates commenced 6 months or less before diagnosis of all UGI cancer; excluding cases where the READ code for UGI cancer diagnosis was uncertain.

UGI Cancer Odds Ratio (95% CI)		
	Women	Men
Excluding bisphosphonates used to treat bone metastases, adjusted for smoking status	1.34 (1.14–1.57)	0.80 (0.61–1.05)
Excluding prescriptions for bisphosphonates commenced 6 months or less before diagnosis of all UGI cancer, adjusted for smoking status	1·30 (1·10–1·53)	0·77 (0·58–1·04)
Excluding cases where the READ code for UGI cancer diagnosis was uncertain, adjusted for smoking	1·34 (1·14–1·56)	0.81 (0·62–1·06)

## Discussion

### Summary of main findings

The results show that female cases of oesophageal cancer were significantly more likely to have been prescribed a bisphosphonate than controls. This effect was more pronounced when we looked at alendronate rather than all bisphosphonates. The risk remained significant after adjusting for to the effect of smoking. There was no significant apparent effect in men although the confidence intervals did not exclude this. There was also no effect on gastric cancer. Adjusting for covariates – dyspepsia, PPI use, BMI, alcohol intake and *H. pylori* status did not alter the result.

Excluding bisphosphonates prescribed for bone metastases; bisphosphonates commenced 6 months or less before the diagnosis of upper GI cancer and cases where there was uncertainty regarding the READ coding of the case did not significantly alter the results.

From the data, 95 out of 4442 female cases of upper GI cancer annually in the UK could be linked to bisphosphonate use (based on an OR of 1·34 for bisphosphonates in women for UGI cancer, 4442 new cases of UGI cancer in women in 2007 and 8·43% of female cases of UGI cancer being prescribed a bisphosphonate).[15]

How our studies differed from other findings

Solomon et al[6] in their response to Wysowski's article used SEER (Surveillance, Epidemiology and End Results) registry data to compare rates of oesophageal cancer in persons receiving oral bisphosphonates with those receiving other medications for osteoporosis and the general incidence rate in the SEER registry. They did not find a difference but, as they admitted, due to the rarity of oesophageal cancer, the confidence intervals were very wide and compatible with an effect as well as no effect.

Nugyen et al[11] performed a nested case-control study examining bisphosphonate use in oesophageal cancer cases and controls in patients with Barrett's oesophagus. The data came from the National Department of Veterans Affairs database. They did not find a significant difference between the groups but the number of cases and controls were relatively small (116 and 696 respectively) and the number of bisphosphonate users in both groups was small which the authors admit limits the power to detect significant differences between the groups.

Abrahamsen and colleagues have reported two cohort studies using data from national registries in Denmark. The first in 2009[5], published as a response to Wysowski's case reports, looked at 13,678 patients with fractures who had used bisphosphonates and compared them with 27,356 controls of the same sex and similar age and fracture type. They found a reduced incidence of oesophageal cancer in the bisphosphonate group and no difference in gastric cancer between the two groups. However, the follow up period was relatively short (2.2 years) and the total number of cases identified small (37 and 48 for gastric and oesophageal cancer respectively). In November 2010 they reported a further study, which compared 30,606 alendronate users with 122,424 matched controls[16]. This time they found a lower risk of gastric cancer in the alendronate group (OR 0.61; 95% CI 0.39–0.97) and no increased risk for oesophageal cancer (OR 0.71; 95% CI 0.43–1.19), however the confidence interval for oesophageal cancer is quite wide and is compatible with an effect as well as no effect. They also found that alendronate users were more likely to have had a recent upper endoscopy.

Vestargaard[17] carried out another cohort study of Danish patients but with significantly larger numbers. 103,562 patients using drugs for osteoporosis were compared with 310,683 age and gender matched controls. They found an excess risk with alendronate and etidronate for oesophageal cancer (RR 2.32 and 2.00 respectively) which was most pronounced for low doses and short duration but still present at higher doses and longer durations.

Chen et al[18] have used claims data from the National Health Insurance Database of Taiwan (NHIRD) to carry out a case control study comparing alendronate prescriptions in 282 cases of oesophageal cancer and 2,811 controls. They found 31.2% of the cases had been prescribed alendronate and 27.1% of the controls which equated to an adjusted OR of 0.61; 95% CI 0.21–1.75 with a p-value of 0.36. The confidence interval, as in other studies, is compatible with a modest effect (e.g. 50% increase or decrease) as well as no effect.

Ho et al[19] recently published another study from Taiwan using the same claims database (NHIRD). This was a large case control study comparing oral bisphosphonate prescribing in 16,204 cases of oesophageal cancer with 64,816 controls. Overall they found bisphosphonates had been prescribed to 7.8% of the cases versus 3.6% of the controls, however they then break the analysis down by frequency of use of bisphosphonates and find a decreasing trend of ORs from rare to regular users of 3.86 to 2.68 and also with time observed and conclude that there is no effect. Unfortunately no confidence intervals are reported and the numbers of frequent and regular bisphosphonate users are very small overall; the authors rely on the basic error of comparing p-values between unequal-sized groups rather than carrying out a valid test.[20] If one looks at the rare user group which has the largest numbers of cases and controls for the three observation periods then the OR's are 3.86, 2.58 and 2,27 for one year, three years and five years respectively with a p value of 0.001.

This last study illustrates the problem of studies on side effects of drugs where the outcome is a rare disease (oesophageal cancer, incidence 9.8∶100,000 UK age standardized rate 2008)[15] and the exposure is also relatively small (average prescribing of bisphosphonates in the UK 4.1% in 2005)[1].

The two previous studies using this data set (GPRD) followed different approaches and had substantially smaller numbers of exposed oesophageal cancer cases (79 Cardwell, 90 Green) than ours. Their confidence intervals are correspondingly wider, and indeed overlap. (Cardwell: adjusted HR 1·07 (95% CI 0·77–1·49); Green: RR1·30 (95% CI 1·02–1·66). In the context of a rare disease, the tiny numerical difference between Hazard Ratio, Risk Ratio and Odds Ratio can be ignored. A formal test for a difference is not possible as the studies draw on the same data. However, there is a substantial overlap of Cardwell & Green's results from 1·02 to 1·49.

In the present study, with 225 exposed oesophageal cancer cases, over twice as many, the OR for oesophageal cancer, for men and women adjusted for smoking, is 1·30 (95% CI 1.21–1·39), which is within the overlap noticed above, but the confidence interval is much narrower.

Possible mechanisms for association of bisphosphonates with oesophageal cancer and why this may vary by gender

The mechanism may be via inflammatory changes to the oesophageal mucosa, well known already as a side effect of bisphosphonates.[21], [22]The apparent lack of an effect in men may be a) due to the much smaller numbers of men (25% of total) prescribed bisphosphonates (and hence the difficulty of demonstrating an effect on a rare disease), however, the significant interaction test strongly suggests that the difference is real; b) the observation that the average age of the men prescribed bisphosphonates (67·8) was less than the women (69·7) and the incidence of UGI cancer increases significantly with age. When we reduced the whole sample size to 25% of the original and re-ran the analysis, the effect in women, although present (OR1.2) was no longer significant (p = 0.2). Women are usually prescribed bisphosphonates for osteoporosis occurring idiopathically whereas men are more likely to be prescribed bisphosphonates for iatrogenically caused osteoporosis, typically from steroids prescribed for chronic obstructive pulmonary disease, or even prophylactically if on high dose steroids for more than three months. Some of the risk factors, apart from prolonged steroid use, for developing osteoporosis – smoking, heavy alcohol intake, anorexia/low BMI, and poor nutrition – also are risk factors for upper GI cancer. Although correcting for these factors in the analysis did not alter the results it may be that as a group women with osteoporosis are at higher risk than men of a similar age for upper GI cancer.

### Strengths and limitations

The main strength of our study is that it is based on one of the most reliable population samples to date and uses electronic patient records rather administrative claims data (Zhang et al[23] have described the problems related to using the latter). It is also based on the largest number of cases giving a relatively precise confidence interval. We were confident from our sample size calculations about being able to detect even quite small differences and that a negative result would be strong evidence against an adverse effect that was important from a public health perspective.

Limitations of our study are that adherence to bisphosphonates was not formally measured (but this is a general difficulty with large anonymised database studies) and although the accuracy of the GPRD cancer codes is likely to be high they were not formally validated. Walker[24] has recently published a study on the accuracy of identification of oesophageal cancer in the GPRD and concluded that while essentially all cases bearing a code for the condition had oesophageal cancer the clinical onset might be significantly earlier than the first code recorded in the GPRD. This could affect cohort studies with short follow up times but not case control studies.

With any retrospective longitudinal study there are also always limitations due to incomplete data recording; prescriptions issued not dispensed/taken; missing data; unmeasured confounders etc.

Oesophageal cancer is rare but often fatal and associated with significant morbidity. Bisphosphonates in general and alendronate in particular are being recommended, by current osteoporosis prevention guidelines, to increasing numbers of men and women in predominantly older age groups. Our data supports a small increased risk of oesophageal cancer in women prescribed bisphosphonates. With a rare disease such as this (incidence of oesophageal cancer in 60–79 age group is 10/100,100) it would need very large prospective cohort studies, with sufficiently long follow up to confirm and clarify the size of the association and none have been published to date, however, Vinogradova et al[25] have recently published a protocol for a large nested case-control study using the Qrisk research database (significantly larger than GPRD) which will look at the association between bisphosphonate prescribing and the 10 most common primary cancers diagnosed in patients between 1996 and 2011.

In the presence of an association and with a plausible mechanism to account for possible causation (irritation of the gastric and oesophageal mucosa) it would be sensible to exercise caution in prescribing bisphosphonates to patients with pre-existing risk factors for upper GI cancer (although unfortunately many of these are also risk factors for developing osteoporosis) and to have a lower threshold for investigating such patients, if on bisphosphonates, should they develop symptoms suggestive of upper GI cancer.

## Supporting Information

Appendix S1 **List of Read/OXMIS codes as evidence of upper GI malignancy.**
(DOCX)

Appendix S2 **Effect of bisphosphonates on UGI cancer for men and women.**
(DOCX)
