# Supplementary material for: Bisphosphonates and Risk of Upper Gastrointestinal Cancer — A Case Control Study Using the General Practice Research Database (GPRD)
Source: PLoS One. 2012 Oct 24;7(10):e47616. doi: 10.1371/journal.pone.0047616 (PMC3480418; doi:10.1371/journal.pone.0047616)
Supplement: Appendix S1 — List of Read/OXMIS codes as evidence of upper GI malignancy. (DOCX) [file pone.0047616.s001.docx]

**APPENDIX S1**

List of Read/OXMIS codes as evidence of upper GI malignancy

| Read Code | Read Term |
| --- | --- |
| B10··00 | Malignant neoplasm of oesophagus |
| B100·00 | Malignant neoplasm of cervical oesophagus |
| B101·00 | Malignant neoplasm of thoracic oesophagus |
| B102·00 | Malignant neoplasm of abdominal oesophagus |
| B103·00 | Malignant neoplasm of upper third of oesophagus |
| B104·00 | Malignant neoplasm of middle third of oesophagus |
| B105·00 | Malignant neoplasm of lower third of oesophagus |
| B106·00 | Malignant neoplasm, overlapping lesion of oesophagus |
| B107·00 | Siewert type I adenocarcinoma |
| B10y·00 | Malignant neoplasm of other specified part of oesophagus |
| B10z·00 | Malignant neoplasm of oesophagus NOS |
| B10z·11 | Oesophageal cancer |
| B11··00 | Malignant neoplasm of stomach |
| B11··11 | Gastric neoplasm |
| B110·00 | Malignant neoplasm of cardia of stomach |
| B110100 | Malignant neoplasm of cardio-oesophageal junction of stomach |
| B110111 | Malignant neoplasm of gastro-oesophageal junction |
| B110z00 | Malignant neoplasm of cardia of stomach NOS |
| B111.00 | Malignant neoplasm of pylorus of stomach |
| B111000 | Malignant neoplasm of prepylorus of stomach |
| B111100 | Malignant neoplasm of pyloric canal of stomach |
| B111z00 | Malignant neoplasm of pylorus of stomach NOS |
| B112·00 | Malignant neoplasm of pyloric antrum of stomach |
| B113·00 | Malignant neoplasm of fundus of stomach |
| B114·00 | Malignant neoplasm of body of stomach |
| B115·00 | Malignant neoplasm of lesser curve of stomach unspecified |
| B116·00 | Malignant neoplasm of greater curve of stomach unspecified |
| B117·00 | Malignant neoplasm, overlapping lesion of stomach |
| B118·00 | Siewert type II adenocarcinoma |
| B119·00 | Siewert type III adenocarcinoma |
| B11y·00 | Malignant neoplasm of other specified site of stomach |
| B11y000 | Malignant neoplasm of anterior wall of stomach NEC |
| B11y100 | Malignant neoplasm of posterior wall of stomach NEC |
| B11yz00 | Malignant neoplasm of other specified site of stomach NOS |
| B11z·00 | Malignant neoplasm of stomach NOS |
| BB55·00 | [M]Linitisplastica |
| BB5C·00 | [M]Gastrinoma and carcinomas |
| BB5C000 | [M]Gastrinoma NOS |
| BB5C011 | [M]G cell tumour NOS |
| BB5C100 | [M]Gastrinoma, malignant |
| BB5Cz00 | [M]Gastrinoma or carcinoma NOS |
