# Supplementary material for: Bisphosphonates and Risk of Upper Gastrointestinal Cancer — A Case Control Study Using the General Practice Research Database (GPRD)
Source: PLoS One. 2012 Oct 24;7(10):e47616. doi: 10.1371/journal.pone.0047616 (PMC3480418; doi:10.1371/journal.pone.0047616)
Supplement: Appendix S2 — Effect of bisphosphonates on UGI cancer for men and women. (DOCX) [file pone.0047616.s002.docx]

**APPENDIX S2**

Effect of bisphosphonates on UGI cancer for men and women (mean number of observations 43,180).

|  | Odds Ratio | P value | 95% confidence interval |
| --- | --- | --- | --- |
| Men and women on bisphosphonates (342 cases, 1227 controls) | 1·13 | 0·06 | 0·99–1·28 |
| Women on bisphosphonates (264 cases, 857 controls) | 1·27 | 0·002 | 1.10-1.47 |
| Men on bisphosphonates(78 cases, 370 controls) | 0·84 | 0·16 | 0·66–1·07 |
